# Significant dose reduction using synchrotron radiation computed tomography: first clinical case and application to high resolution CT exams

**DOI:** 10.1038/s41598-018-30902-y

**Published:** 2018-08-21

**Authors:** H. Labriet, C. Nemoz, M. Renier, P. Berkvens, T. Brochard, R. Cassagne, H. Elleaume, F. Estève, C. Verry, J. Balosso, J. F. Adam, E. Brun

**Affiliations:** 1 Centre Hospitalier Universitaire de Grenoble, avenue Maquis du Grésivaudan, 38700 La Tronche, France; 2 Rayonnement Synchrotron et Recherche Médicale, EA 7442, Université Grenoble-Alpes, Grenoble, France; 3 European Synchrotron Radiation Facility, 71 avenue des Martyrs, 38043 Grenoble, France

## Abstract

Since the invention of Computed Tomography (CT), many technological advances emerged to improve the image sensitivity and resolution. However, no new source types were developed for clinical use. In this study, for the first time, coherent monochromatic X-rays from a synchrotron radiation source were used to acquire 3D CTs on patients. The aim of this work was to evaluate the clinical potential of the images acquired using Synchrotron Radiation CT (SRCT). SRCTs were acquired using monochromatic X-rays tuned at 80 keV (0.350 × 0.350 × 2 mm^3^ voxel size). A quantitative image quality comparison study was carried out on phantoms between a state of the art clinical CT and SRCT images. Dedicated iterative algorithms were developed to optimize the image quality and further reduce the delivered dose by a factor of 12 while keeping a better image quality than the one obtained with a clinical CT scanner. We finally show in this paper the very first SRCT results of one patient who received Synchrotron Radiotherapy in an ongoing clinical trial. This demonstrates the potential of the technique in terms of image quality improvement at a reduced radiation dose for inner ear visualization.

## Introduction

Computed Tomography (CT) - with 62 million exams performed each year in the USA - is the most common clinical examination using the principle of X-ray attenuation^1^ and the most frequently used 3D medical diagnostic tool yielding to an increased overall radiation dose delivered to patients for medical purposes. Recent studies estimated that 3% of cancers may be attributable to diagnostic CT^2^ and that a cumulative dose of 50 mGy in childhood triples the risk of brain tumor and leukemia^3^.

Since its invention and the seminal work performed by Hounsfield and Cormack^4^, many technological advances emerged to improve the image sensitivity and the spatial resolution^5^. However, to the best of our knowledge, no new source type was developed for clinical use despite the current limitations of conventional bremsstrahlung x-ray sources, such as low flux, strong divergence, broad energy spectrum and associated artifacts, drift or heating problems.

In the past two decades synchrotron radiation has shown its potential in biomedical imaging^6^ mainly in mammography^7^ or in musculo-skeletal applications^8^. Synchrotron Radiation Computed Tomography (SRCT) became then the gold standard technique for measuring 3D linear attenuation coefficients^9^. However, until now all these biomedical studies were limited to human anatomical pieces or to preclinical studies on small animals.

In this work we first evaluate both quantitatively and qualitatively the performance of SRCT on quality assurance phantoms to show that significant dose reduction can be achieved with improved image quality. The second part of the paper is devoted to the clinical SRCT results on a patient with a focus on high resolution inner ear exam at a low dose.

## Methods

The SRCT images were acquired in the context of patient positioning in an on-going phase I/II contrast enhanced synchrotron stereotactic radiotherapy (SSRT) clinical trial performed at the European Synchrotron Radiation Facility (ESRF) biomedical beamline (ID17).

The procedures followed in the present study were in accordance with the ethical standards of the responsible committee on human experimentation: the regional ethical committee designed by the French ministry of health approved our protocol before any patient recruitment (comité de protection des personnes sud est V, CS10217, 38043 Grenoble cedex 9, France), refs CPP-10CHUG09 and 2010-A00773-36. Each recruited patient signed an informed consent form prior to their inclusion in the present study.

**Figure 1:**
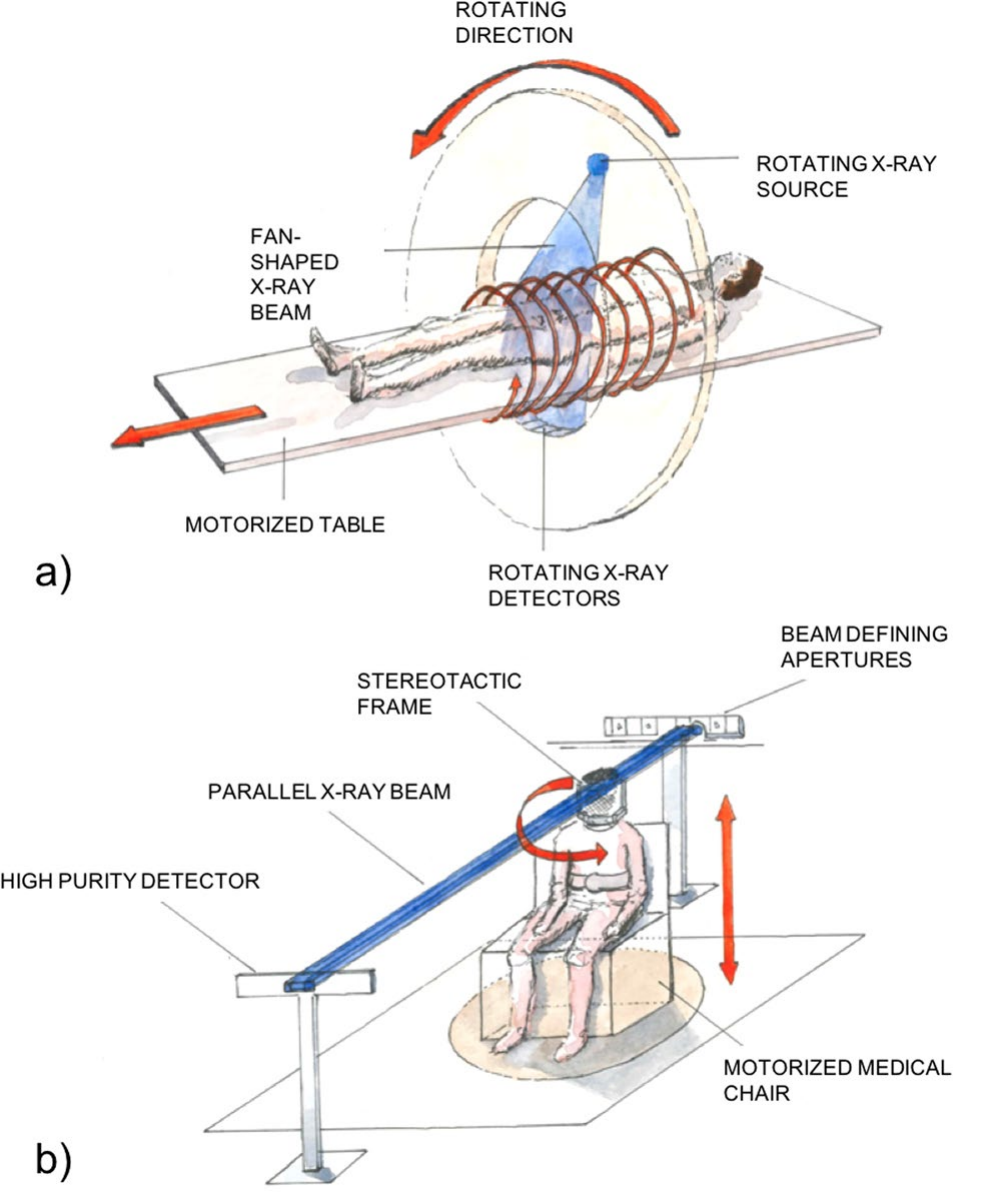
Presentation of the clinical (**a**) and the synchrotron radiation (**b**) computed tomography set-up. At the hospital a cone or fan beam and the detectors are rotating around the patients whilst at the synchrotron the patient is sat on a moving and rotating chair.

The experimental set-up used for SRCT and its comparison with a conventional X-ray CT scanner are shown in Fig. 1.

In a conventional clinical set-up, both the source and the detector rotate around the patient. In SRCT, the patient is rotated on an axis perpendicular to the beam propagation. The SRCT X-ray beam is monochromatic with an energy tunable between 30 and 120 keV (1‰ bandwidth) and quasi-parallel (<1mrad divergence), whilst commercial CT scanners use fan or cone shaped polychromatic beams. The quasi-parallel beam is indeed unique and exhibits advantages over the fan or cone shaped beam. For example, interpolation is not required for the tomographic reconstruction algorithm. The synchrotron storage ring is a 6 GeV machine and the x-rays are produced using two multipole wigglers located 150 m from the patient positioning system. An electron ring current of about 200 mA was used for our experiment. The focal source size is 60 µm (full half width maximum). The beam at the patient level is 150 mm wide, 2 mm height and can be considered as parallel.

For the sake of comparison conventional images were acquired on a recent diagnostic scanner (GE Healthcare Revolution, Chicago, USA) available from the local neuroradiology department. The hospital CT operated at 120 kVp in helical mode (pitch = 0.53) with fixed mAs, a FOV of 22.5 cm and a 512 × 512 pixels matrix (0.44 mm pixel size) for the whole study.

Two imaging protocols were used on the conventional CT:

- a standard head CT protocol: 200 mAs, 1.25 mm slice thickness, CTDI = 33 mGy.
- an inner ear CT protocol, to obtain the highest resolution achievable for diagnostic routine exams: 350 mAs CTDI = 183 mGy.

The dose for both clinical CT and SRCT was measured using a calibrated PTW (Freiburg Germany) CT ionization chamber (PTW 31336) within a head phantom, standard head CTDI, (IBA Dosimetry, Germany) in a cylindrical volume of 16 cm in diameter with a 1 cm scan in height.

The SRCT configuration used a 150 mm wide, 2 mm height 80 keV X-ray beam^10^. The detector was positioned 6 m downstream of the patient. A high purity germanium detector composed of 431 pixels (350 µm in width) was used in current integration mode (2 ms integration time per projection). The radiotherapy photon flux was reduced by adding PolyMethyl MethAcrylate (PMMA) attenuators of various thicknesses (0.5 to 32.5 cm). A so-called half-acquisition mode^11^ was used to raise the reconstruction field of view to 28 cm. We acquired 1024 projections around 360 degrees at the rotation speed of 87.5 degrees/s. Due to the laminar shape of the beam the imaging acquisition protocol consists of several rotations followed by vertical movements in order to scan the patient region of interest. The total imaging acquisition time for the patient was 123 s for 30 slices.

For this study two imaging protocols were used at the ESRF:

- The low dose imaging protocol: slice thickness 0.625 mm, 18 cm PMMA attenuators. We then virtually removed 2/3 of the projections and present 340 projections reconstructions with CTDI = 14.8 mGy.
- The SSRT clinical trial imaging protocol using no modifications from the treatment beam geometry to the imaging beam, except flux reduction: slice thickness 2 mm, 14.5 cm PMMA attenuators, CTDI = 93 mGy.

A Catphan 504 (PhantomLab, Salem, NY, USA) was used to compare quantitatively the SRCT images with the gold standard clinical CT. The different acquisition protocols are summarized in Table 1. Raw images were first reconstructed by a simple filtered back projection algorithm^12^ and processed using the Normalized Metal Artifact Reduction^13^ approach to remove artifacts caused by patient positioning seeds. 340 projections were chosen as approximatively 1/3 of the original number of projections, and being a value mathematically suitable for the EST. At 340 projections, the images (patient and phantom) still exhibits an improved quality when compared to conventional CT (as detailed in the results section). Using a lower number of projections start to impair the image quality.

**Table 1:** Acquisition and CTDI parameters of the various scans performed in this comparative study.

Acquisition protocols		Energy	PMMA thickness	Slice thickness	Pixel size	Number of projections	Dose CTDI
			(cm)	(mm)	(mm)	(−)	(mGy)
Conventional clinical CT	Standard head (patient)	120 kVp	n.a.	1.25	0.44	n.a	33
		250 mA					
	Inner ear (phantom)	120 kVp	n.a.	0.625	0.44	n.a	183
		350 mA					
SR-CT	Patient acquisition	80 keV	14.5	2	0.35	1024	93
		191 mA					
	‘Low dose’ (phantom)	80 keV	18	0.625	0.35	340	14.8

In order to further increase the SNR in soft tissues whilst keeping a high image quality in the bone tissues, we implemented a multi-component reconstruction procedure. For each sinogram, we process bone and soft tissue components separately with adapted filters and reconstruction parameters. Starting from the raw reconstruction, i.e., standard Filtered Back Projection, we segment each tissue by thresholding. After segmentation, a projection operator is used to obtain the radiographs for each tissue. A low-pass filter is then applied in the Fourier domain for each radiograph. We empirically adapted for each tissue the kernel sizes according to its softness. A classical Filtered Back Projection algorithm is then applied to obtain each tissue CT images. In order to reproduce the entire volume, the CT data are then merged in one single volume using the masks generated by the original segmentation.

**Figure 2:**
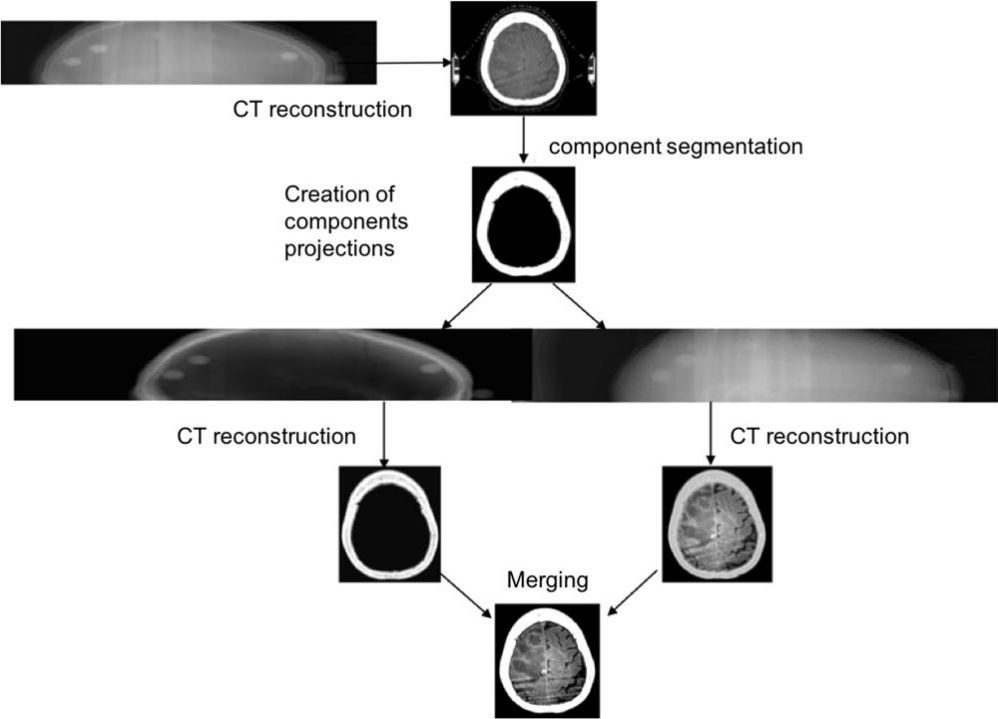
Scheme of the reconstruction algorithm for improving the SNR on SRCT images.

Figure 2 presents a flowchart of this iterative image reconstruction algorithm.

To evaluate the possibility of further dose reduction, we virtually minimized the number of projections (340 instead of 1024) and used an iterative reconstruction algorithm: Equally Sloped Tomography (EST) which has shown high potential when the number of projections is low^7,14,15^. For further details of the EST method please refer to^7^. Altogether SRCT with EST reconstruction achieve a CTDI of 14.8 mGy.

The Signal to Noise Ratio (SNR) (Eq. 1) and Contrast to Noise Ratio (CNR) (Eq. 2) were measured in the homogenous and low contrast modules, using the following definitions:

1 $${\rm{SNR}}=\frac{\bar{\mu }}{{\rm{\sigma }}}$$

2 $${\rm{CNR}}=\frac{\overline{{\mu }_{1}}-\overline{{\mu }_{2}}}{{\rm{\sigma }}}$$

where $\bar{{\rm{\mu }}}$ is the average Hounsfield value in homogeneous ROI and $\sigma$ is the standard deviation in the considered ROI; ${{\rm{\mu }}}_{1}$ and ${{\rm{\mu }}}_{2}$ are defined as average Hounsfield values in two different homogenous zones. These measurements of CNR and SNR were made on 4 ROIs in the Catphan image. The spatial resolution was estimated visually using the MTF (Modulation Transfer Function) module by a trained x-ray examiner.

Finally, one patient bearing a brain metastasis close to the inner ear has been imaged during the positioning and contrast agent uptake verification procedure in the framework of the SSRT clinical trials. The imaging acquisition parameters, such as energy, spatial resolution, slice thickness and flux, were defined by the radiotherapy trial constraints and thus could not be optimized, as it would be the case for an imaging clinical trial.

## Results

**Figure 3:**
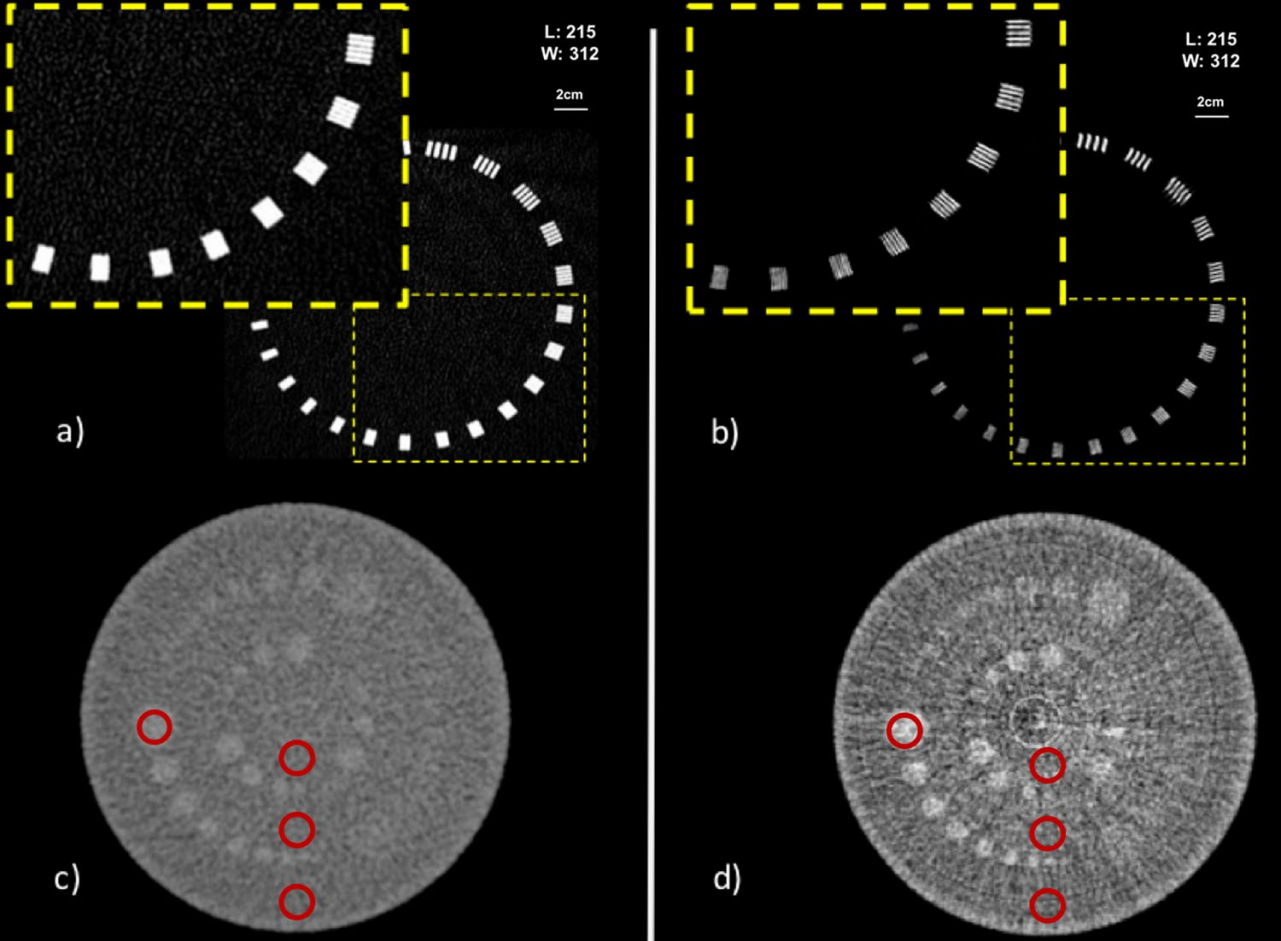
Images acquired on the Catphan 504 phantom. MTF module on the top (**a** and **b**) and low contrast module on the bottom (**c** and **d**) (**a** and **c**) are acquired at the hospital with the inner ear protocol (CTDI 183mGy). (**b** and **d**) were acquired at the ESRF with low dose reconstruction (CTDI 14.8mGy). The region of interest where the image quality parameters were measured (12 mm diameter) are represented with red circles.

Figure 3 shows both the high resolution MTF and the low contrast modules reconstruction for two acquisitions, inner ear conventional CT and low dose SRCT reconstructed by the EST method using 340 projections. From these images, it is clear that SRCT at a reduced dose outperforms the state-of-the-art clinical CT in term of image quality for the inner ear protocol.

Figures a and b present the resolution module of the Catphan with a zoom in the harder part to distinguish. Images c and d present the low contrast module with the 3 groups of contrast. The improvement of the contrast between the different inserts is visible in Fig. 3(c and d). The catphan phantom has a low contrast module with supra-slice and subslice contrast targets. The low contrast targets have different diameters and contrasts. The subslice target diameters range from 3.0 mm to 9.07 mm. The contrast of the 3 groups are 1%, 0.5% and 0.3% difference in attenuation with the background slice. Two groups of inserts can be seen in both modalities but in the SRCT image a difference can be noted between the 0.5% and the 1%. The high resolution module with 21 line pair per cm gauge of the catphan allows evaluation of the resolution obtained. This module section has a set of high resolution patterns in pair of line per cm. Figure 3(a and b) demonstrate the improved resolution achieved by the SRCT. It is particularly interesting to note that the low dose reconstruction succeeds in improving the resolution by 4 line pairs per mm (from 11 to 15) in comparison with the inner ear clinical protocol whilst the dose to the sample has been reduced by a factor 12.

Table 2 summarizes the quantitative results obtained on the Catphan phantom for the central background ROI (worth scenario for SNR or CNR considering the artifacts seen on Fig. 3). Both resolution and signal to noise ratio are greater for the two SRCT acquisitions when compared to the clinical acquisitions for each ROI described on Fig. 3. The SNR is multiplied by at least a factor 20 between the two high resolution protocols. The CNR is slightly improved.

**Table 2:** Contrast to Noise Ratio, Signal to Noise Ratio and resolution for the different acquisition protocols *(central background ROI)*. EST: Equally Sloped Tomography.

Acquisition protocols		CNR	SNR	MTF (pl/mm)	CTDI (mGy)
Conventional CT	Standard head	1.94	13.02	10	33
	Inner ear	1.26	10.33	11	183
SR-CT	Patient acquisition	4.46	540.06	15	93
	‘Low dose’ (340 projections, EST)	1.91	217.65	15	14.8

CNR: Contrast to Noise Ratio. SNR: Signal to Noise Ratio. MTF: Modulation Transfer Function. CTDI: Computed Tomography Dose Index.

**Figure 4:**
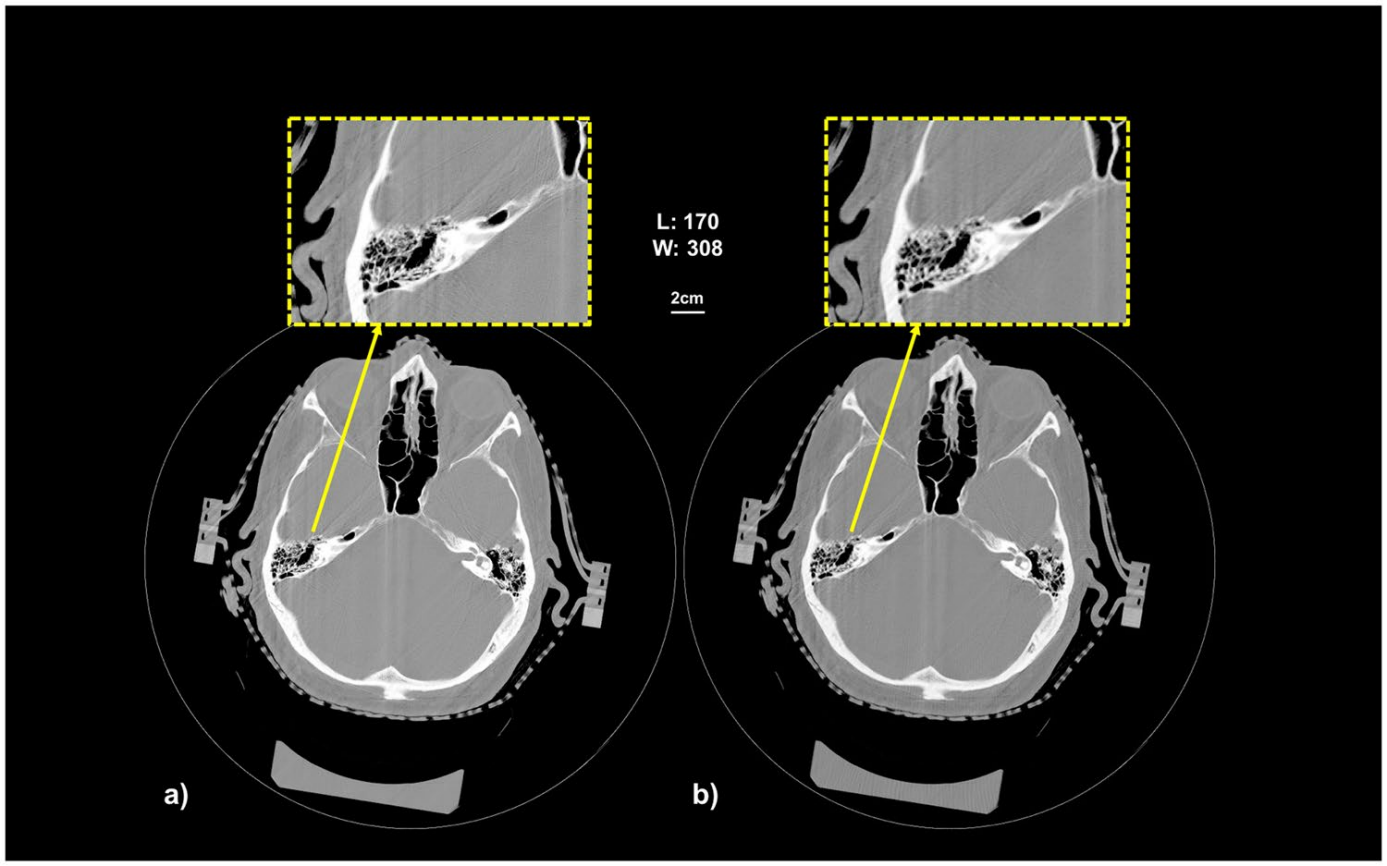
Patient SRCT slices reconstructed with 1024 (**a**) projections using the EST algorithm; The image (**b**) was reconstructed using 340 projections over the available 1024 projections.

Figure 4(a and b) show the SRCT reconstructed image of the inner ear of one patient, using 1024 projections and 340 projections, respectively. The patient was immobilized in a stereotactic frame using a thermoform mask (Brainlab, Munich, Germany). We note that the virtual dose reduction can be achieved without image quality deterioration. The bone structure is well depicted in the both images. If we zoom in the inner ear structure, we can appreciate the resolution achieved in the temporal bones. We can clearly see the common cystic cavity, the internal acoustic meatus and all the air cells in the mastoid bone for example. The image quality is improved even if the number of projection is reduced. Please note that the slice thickness of the images is 2 mm due to the radiotherapy trial safety constraint. It is for this reason that the images of the inner ear do not show a substantial improvement if compared to a standard clinical inner ear CT. Nevertheless, the slice thickness in the SRCT protocol can be further reduced without increasing the radiation dose as we demonstrated for the phantom. Moreover, we can expect a further reduction by lowering the photon flux, which was not possible on the patient within the clinical trial.

## Discussion and Conclusion

Imaging plays an increasing key role in the diagnosis and in the management of the numerous head and neck diseases. X-ray CT is the most commonly used examination and corresponds to the most important part of the population’s artificial ionizing radiation exposure. Although the risks associated withone exam remain low, the increased lifetime can lead to a public health hazard in the future^2^. During the last few years, the number of diagnostic CT examinations performed has increased dramatically. Many efforts have been made by manufacturers to decrease the dose in a single exam by using iterative reconstruction algorithms and/or more efficient detectors. However, to the best of our knowledge, no significant attempt has been made to use other types of X-ray sources on patients.

Conventional CT is limited for the detection and visualization of small variations in brain tissues mass densities, due to the small differences of mass attenuation coefficients between the soft tissues. That’s why conventional CT generally requires the use of iodine as contrast agent to improve visualization. The problem in using contrast agents is not only limited to CT exams, but to MRI as well, which often requires the injection of gadolinium-based contrast agents. Recent discoveries showed that some free gadolinium, which is highly toxic, persists in the brain of patients after several injections^16^. Moreover, it has been recently shown that MRI combined with the injection of gadolinium may lead to additional DNA double strand breaks^17^. Developing new types of diagnostic imaging modalities like SRCT that do not always require the use of contrast agents and operate at a lower radiation dose appears thus as an option to be seriously considered in diagnostic radiology.

The aims of this study were to evaluate the performance and the potential of synchrotron radiation CT using tunable monochromatic low divergence X-rays. This study was performed on a quality assurance phantom as well on a patient involved in an ongoing radiotherapy program clinical trial at the ESRF.

According to the IRSN (“Institut de Radioprotection et de Sureté Nucléaire”, the French Regulator) the dose routinely delivered in hospitals for a head diagnosis CT is 65 mGy^18^. Our study shows that the delivered doses could be significantly reduced while increasing the global image quality thanks to the combination of small pixel size, high spatial coherence, monochromaticity, and negligible scatter detection due to the long distance between the patient and the detector.

Altogether, if we combine the high performances of the synchrotron source, the EST reconstruction and the flux reduction prospect, we would obtain a dose reduced by a factor 12 when compared to the state-of-the-art clinical CT. This is of particular interest for high-resolution head & neck CTs. The use of such an iterative reconstruction algorithm is also possible outside the synchrotron world and can be applied on clinical datasets^19^.

Even if the trial has not been designed specifically for imaging, these SRCT data sets on patients are the first ever obtained on humans with a synchrotron source and clearly show the potential of SRCT in clinical high resolution imaging at a reduced radiation dose. Performing CT using a synchrotron source is thus feasible in clinics, even if scanning the brain region. It allows to resolve complex bone structures at a lower dose than clinical routine protocols. This is by itself a significant added value x-ray CT imaging.

In the past two decades synchrotron radiation has shown its potential in biomedical imaging^6^: in mammography, for the diagnosis of human breast cancers^7,20^, on human knee^8^ and other types of tissue^6^. SRCT became then the gold standard technique for measuring 3D linear attenuation coefficients^9^. Until now these biomedical studies were limited to human anatomical samples or to preclinical studies on small animals^9,21^. The SYRMA-CT/3D^22^ (SYnchrotron Radiation- MAmmography-Computed Tomography/three-Dimensional) collaboration aims to set-up the first clinical trial of PhC breast CT with SR. This phase 1 clinical trial on mammography has been set-up in Trieste in 2016 with the aim of resolving questionable diagnosis after the hospital exam, based on conventional mammography and ultrasound. Moreover, as the radiotherapy clinical trial associated to the current study requires the injection of 200–250 mL of iodinated contrast agent, it is completely safe to proceed with SR-CT after injection of a quite large amount of contrast agent as it could be required by osteoarticular or oncology studies. Phase contrast CT imaging techniques are promising as the natural contrast is enhanced. This raises the question if a proper diagnosis could be performed without injection of contrast agent and thus avoid adverse side effects. However further SR-CT studies should be performed on patients, with and without contrast administration, with clear endpoints in terms of sensitivity and specificity assessment to reply to that complex question. It is too early to make any statement and the answer might be pathology or anatomy dependent.

All these results encourage imaging with new types of X-ray sources where monochromaticity and coherence are made possible to spread it outside the synchrotron world where beamtime access could be a serious limitation. New source types which are more compact than a synchrotron are currently in their development phase in this direction^23^, for a wider development of the technique in an environment closer to the hospital one. This proof of concept study allows us to envisage a wider spread use of highly coherent X-ray sources because the results show a higher image quality at a lower radiation dose deposition. A transfer of the technique to other sources is an essential step to consider a clinical application. Currently new compact X-ray sources such as ThomX^23^ are in development. The goal of the ThomX project is to design and build a facility with a flux between 10^11^–10^13^ γ/s in the hard X-ray range and the photon energy tunability will provide a Compton edge that can be set between 50 and 90 keV. Such sources have synchrotron-like characteristics in terms of brightness and coherence^24^ and will provide a much larger accessibility to the technique.

The study presented in this paper shows the very first live human CT dataset obtained on a synchrotron source and will undoubtedly pave the way of a wider development of monochromatic and coherent x-ray imaging techniques for various medical applications such as oncology or osteo-articular diseases.

## Data Availability

All data generated or analysed during this study are included in this published article.

## References

[1] Brenner DJ, Hall EJ (2007). Computed tomography–an increasing source of radiation exposure. N. Engl. J. Med..

[2] De González AB, Darby S (2004). Risk of cancer from diagnostic X-rays: Estimates for the UK and 14 other countries. Lancet.

[3] Pearce MS (2012). Radiation exposure from CT scans in childhood and subsequent risk of leukaemia and brain tumours: A retrospective cohort study. Lancet.

[4] Hounsfield, G. N. Computerized transverse axial scanning (tomography): Part I. Description of system. *Br J Radiol* **46** (1973). 10.1259/0007-1285-46-552-1016 4757352

[5] Hsieh, J. *Computed Tomography, Second Edition*. 10.1117/3.817303 (SPIE, 2009).

[6] Bravin A, Coan P, Suortti P (2013). X-ray phase-contrast imaging: from pre-clinical applications towards clinics. Phys. Med. Biol..

[7] Zhao Y (2012). High-resolution, low-dose phase contrast X-ray tomography for 3D diagnosis of human breast cancers. Proc. Natl. Acad. Sci. USA.

[8] Horng A (2014). Cartilage and soft tissue imaging using X-rays: propagation-based phase-contrast computed tomography of the human knee in comparison with clinical imaging techniques and histology. Invest. Radiol..

[9] Elleaume, H., Charvet, A. M., Corde, S., Estève, F. & Bas, J. F. L. Performance of computed tomography for contrast agent concentration measurements with monochromatic x-ray beams: comparison of K-edge versus temporal subtraction. *Phys. Med. Biol.* **47**, 3369–85 (2002). 10.1088/0031-9155/47/18/307 12375826

[10] Edouard M (2010). Treatment plans optimization for contrast-enhanced synchrotron stereotactic radiotherapy. Med. Phys..

[11] Wang G (2002). X-ray micro-CT with a displaced detector array. Med. Phys..

[12] Mirone A, Brun E, Gouillart E, Tafforeau P, Kieffer J (2014). The PyHST2 hybrid distributed code for high speed tomographic reconstruction with iterative reconstruction and a priori knowledge capabilities. Nucl. Instruments Methods Phys. Res. Sect. B Beam Interact. with Mater. Atoms.

[13] Meyer E, Raupach R, Lell M, Schmidt B, Kachelriess M (2010). Normalized metal artifact reduction (NMAR) in computed tomography. Med. Phys..

[14] Scott MC (2012). Electron tomography at 2.4-ångström resolution. Nature.

[15] Chen C-C (2013). Three-dimensional imaging of dislocations in a nanoparticle at atomic resolution. Nature.

[16] Robert P (2015). T1-Weighted Hypersignal in the Deep Cerebellar Nuclei After Repeated Administrations of Gadolinium-Based Contrast Agents in Healthy Rats. Invest. Radiol..

[17] Reddig A (2015). Analysis of DNA Double-Strand Breaks and Cytotoxicity after 7 Tesla Magnetic Resonance Imaging of Isolated Human Lymphocytes. PLoS One.

[18] Irsn. Analyse des données relatives à la mise à jour des niveaux de référence diagnostiques en radiologie et en médecine nucléaire - Bilan 2011–2012 (2011).

[19] Fahimian BP (2013). Radiation dose reduction in medical x-ray CT via Fourier-based iterative reconstruction. Med. Phys..

[20] Ruiz-Gonzalez, Y. *et al*. Objective measurements of image quality in synchrotron radiation phase-contrast imaging versus digital mammography. *Int. J. Comput. Assist. Radiol. Surg*. 10.1007/s11548-015-1237-7 (2015). 10.1007/s11548-015-1237-7 26092659

[21] Xi Y, Lin X, Yuan F, Yang G-Y, Zhao J (2015). High-Resolution and Quantitative X-Ray Phase-Contrast Tomography for Mouse Brain Research. Comput. Math. Methods Med..

[22] Longo R (2016). Towards breast tomography with synchrotron radiation at Elettra: first images. Phys. Med. Biol..

[23] Variola A (2014). The ThomX project status. WEPRO.

[24] Jacquet M (2016). Potential of compact Compton sources in the medical field. Phys Med..

